# Flexible Flat Foot, Short Tendo-Achilles, and Altered Gait

**DOI:** 10.7759/cureus.21983

**Published:** 2022-02-07

**Authors:** Ajith Malige, Hannah Chang, Xochitl Mellor, Divya Talwar, Richard S Davidson

**Affiliations:** 1 Orthopedic Surgery, St. Luke's University Health Network, Bethlehem, USA; 2 Orthopedic Surgery, Children's Hospital of Philadelphia, Philadelphia, USA

**Keywords:** pediatric, hip, back, pain, gait, achilles, short, feet, flat

## Abstract

Background

Pediatric patients often present with vague complaints involving the anterolateral foot and ankle, the medial knee, the lower back, and the hip. In our experience, closer examination of these patients reveals a constellation of symptoms that involve pathology in the ankle, knee, back, and hip. This study aimed to detail the identification and treatment of patients with the triad of flexible flat feet, tight Achilles complex, and altered gait, and their clinical course over time.

Methods

All patients of age 18 years or younger who presented to our urban academic center outpatient clinic with foot, ankle, patellar, low back, or hip pain or pathology were included. Patients with identified tarsal coalitions, accessory naviculars, malalignment syndrome, bone cysts or tumors, soft tissue tumors, arthropathy, neuropathy, infection, limb length inequality, patellar subluxation or dislocation, or spinal pathology were excluded. For each patient, demographic information, symptom description, treatment, and clinical course, including the Foot and Ankle Outcome Score (FAOS) was recorded.

Results

A total of 62 patients were included in our study. Patients mostly presented with low back pain (n=24, 38.7%), medial patellar pain (n=36, 58.1%), anterolateral ankle pain (n=35, 56.5%), and vague foot pain (n=13, 21.0%). Overall, 53.8% of patients with back pain at the initial visit, 35.0% of patients with knee pain, 44.4% of patients with ankle pain and 80.0% of patients with foot pain improved at final follow up. While patient-reported sports and recreation subscale scores demonstrated a significant improvement at the final follow-up compared to baseline scores (p=0.02), all other scores did not significantly differ compared to baseline scores. At final follow-up, 12 of 26 (46.2%) patients reported being able to return to play in their desired sport.

Conclusion

Complaints of back, hip, knee, ankle, or foot pain in the pediatric population can be early markers for a constellation of conditions that include low back pain, flexible flat feet, Achilles contracture, and altered gait due to increased lateral subluxation of the patella and hip flexion.

## Introduction

Pediatric patients often present with vague multiple complaints involving the anterolateral foot and ankle, the medial knee, the lower back, and the hip. Kosashvili et al. studied 92,000 Israeli adolescent military recruits and discovered a link between flat foot severity and the prevalence of anterior knee pain and intermittent low back pain, with moderate and severe flat foot being associated with nearly double the rate of knee and low back pain [1]. In 2010, a follow-up study found a 15.3% incidence of flexible flatfoot among 97,000 Israeli military recruits, with these patients also having a higher rate of anterior knee pain compared to those with flexible pes planus [2]. A third study found that patients with flat feet had 1.3 times the odds of knee pain and 1.4 times the odds of cartilage damage in adults [3].

Closer examination of patients with any of the abovementioned complaints may lead to the discovery of a constellation of symptoms that involve pathology in the ankle, knee, back, and hip. This stems from a physical triad of flexible flat feet, contracture of the gastrocnemius-soleus muscle complex, and altered gait resulting in a change in kinematics manifesting as lower back pain, lateral subluxation of the patella, and hip pain. An understanding of flat foot and the pain triad may contribute to improved patient outcomes, as the presence of flat foot diminishes the quality of life and foot function [4]. Flat foot also often presents with anterolateral foot and ankle pain secondary to impingement [5], a tight Achilles tendon complex that may benefit from stretching or surgical lengthening [5,6], lateral patellar subluxation resulting in medial facet patellar pain, lower back pain, and hip pathology resulting in altered gait [7].

While the studies above have examined adults and adolescents, the literature in pediatric patients is severely lacking, especially with regards to the described triad. This study aimed to describe, define, and assess the identification and treatment of patients with the triad of flexible flat feet, tight Achilles complex, and altered gait as well as their clinical course over time. We hypothesize that with proper early diagnosis and focused treatment, patients with this triad will clinically improve over time.

## Materials and methods

Approval for our observational cohort study was obtained from Children's Hospital of Philadelphia Institutional Review Board (approval #13-009982). All patients 18 years of age and younger over a two-year period who presented to our urban academic medical center outpatient clinic were eligible to be part of our study and were retrospectively reviewed. All patients who presented with foot, ankle, patellar, low back, or hip pathology pain were included. In addition, all patients who presented with diagnoses of flexible pes planus and ankle dorsiflexion of 10 degrees or less, regardless of the presence of hip or knee pathology leading to gait instability, were also included. A combination of physical examination findings (inspection and deformity correction), as well as radiographs, were utilized for making a diagnosis of flexible pes planus in all age groups [8,9]. Patients older than 18 years of age and those with identified tarsal coalitions, accessory naviculars, malalignment syndrome, bone cysts or tumors, soft tissue tumors, arthropathy, neuropathy, infection, limb length inequality, patellar subluxation or dislocation, or spinal pathology were excluded. For each patient, demographic information, symptom description, treatment, and clinical course were recorded. Each participant completed the Foot and Ankle Outcome Score (FAOS) during the initial clinical visit and each subsequent follow-up visit. Each patient who did not have a clinical follow-up two years after their initial evaluation was called to complete a phone visit to collect information about their clinical course and symptomology as well as have them complete a FAOS questionnaire.

Data were analyzed using simple descriptive statistics and statistical significance tests (one-way ANOVA and Wilcoxon signed-rank tests) as appropriate to determine any significant changes at each clinic visit compared to baseline (SPSS Version 23 {Armonk, NY: IBM Corp.} for Windows). For all analyses, p<0.05 denotes statistical significance.

## Results

In total, 62 patients were included in our study. Most of our cohort was female (n=45, 72.6%), between the age of 12 years and 15 years (n=42, 67.7%), and had a BMI less than or equal to 25.0 (n=42, 67.7%). In total, 19 (30.6%) patients initially did not present with pain while four (6.5%) presented with pain in one location, seven (11.3%) in two locations, 19 (30.6%) in three locations, 11 (17.7%) in four locations, and two (3.2%) in five locations (Table 1). Four (6.5%) patients reported that their symptoms started after an acute injury, 29 (46.8%) reported symptoms that presented gradually over time with participation in sports, and 22 (35.5%) patients reported symptoms that presented gradually without participation in sports (the remaining seven did not specify). Twenty-four patients presented for a follow-up appointment, eight for two follow-up appointments, and four for three follow-up appointments. In total, 26 patients were reachable for a final follow-up (either in person or by phone call) after two years from the initial presentation.

**Table 1:** Demographic characteristics of our sample population.

Demographic		Total Locations of Pain						Total
		0	1	2	3	4	5	
Gender	Male	7 (11.3%)	1 (1.6%)	0 (0.0%)	6 (9.7%)	3 (4.8%)	0 (0.0%)	17 (27.4%)
	Female	12 (19.4%)	3 (4.8%)	7 (11.3%)	13 (21.0%)	8 (12.9%)	2 (3.2%)	45 (72.6%)
Age	≤ 11 years	7 (11.3%)	1 (1.6%)	1 (1.6%)	3 (4.8%)	2 (3.2%)	0 (0.0%)	14 (22.6%)
	12-13 years	7 (11.3%)	2 (3.2%)	2 (3.2%)	7 (11.3%)	3 (4.8%)	2 (3.2%)	23 (37.1%)
	14-15 years	5 (8.1%)	0 (0.0%)	3 (4.8%)	6 (9.7%)	5 (8.1%)	0 (0.0%)	19 (30.6%)
	≥ 16 years	0 (0.0%)	1 (1.6%)	1 (1.6%)	3 (4.8%)	1 (1.6%)	0 (0.0%)	6 (9.7%)
Body Mass Index (BMI)	≤ 25.0	11 (17.7%)	4 (6.5%)	4 (6.5%)	13 (21.0%)	9 (14.5%)	1 (1.6%)	42 (67.7%)
	25.1-30.0	2 (3.2%)	0 (0.0%)	1 (1.6%)	2 (3.2%)	0 (0.0%)	1 (1.6%)	6 (9.7%)
	30.1-35.0	2 (3.2%)	0 (0.0%)	1 (1.6%)	1 (1.6%)	1 (1.6%)	0 (0.0%)	5 (8.1%)
	≥ 35.1	0 (0.0%)	0 (0.0%)	0 (0.0%)	1 (1.6%)	0 (0.0%)	0 (0.0%)	1 (1.6%)
	Not Recorded	4 (6.5%)	0 (0.0%)	1 (1.6%)	2 (3.2%)	1 (1.6%)	0 (0.0%)	8 (12.9%)
Total		19 (30.6%)	4 (6.5%)	7 (11.3%)	19 (30.6%)	11 (17.7%)	2 (3.2%)	62 (100.0%)

**Figure 1:**
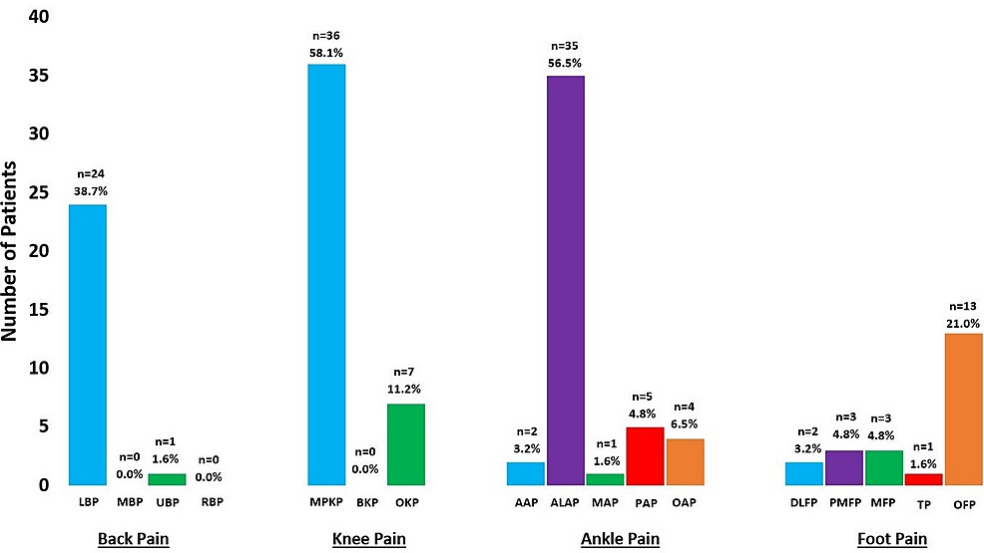
Description of patient's pain symptoms (back, knee, ankle, and foot pain). LBP: lower back pain; MBP: middle back pain; UBP: upper back pain; RBP: radiating back pain; MPKP: medial patellar knee pain; BKP: back of knee pain; OKP: other knee pain; AAP: anterior ankle pain; ALAP: anterolateral ankle pain; MAP: medial ankle pain; PAP: posterior ankle pain; OAP: other ankle pain; DLFP: dorsolateral foot pain; PMFP: plantar midfoot pain; MFP: metatarsal foot pain; TP: toe pain; OP: other foot pain

Thirty-two (51.6%) patients complained of back pain at initial evaluation, two (3.2%) complained of hip pain, 41 (66.1%) of knee pain, 39 (62.9%) of ankle pain, 13 (21.0%) of foot pain, and two (3.2%) of pain at other sites. Patients were not asked to detail their hip pain. Patients mostly presented with lower back pain (n=24, 38.7%), medial patellar pain (n=36, 58.1%), anterolateral ankle pain (n=35, 56.5%), and other foot pain (n=13, 21.0%) (Figure 1). Thirty-five (56.5%) patients underwent physical therapy (Achilles and quadriceps stretching and strengthening), 13 (21.0%) used orthotics, nine (14.5%) used bracing (ankle brace or CAM Walker boot), and two (3.2%) underwent surgery (osteotomies).

**Figure 2:**
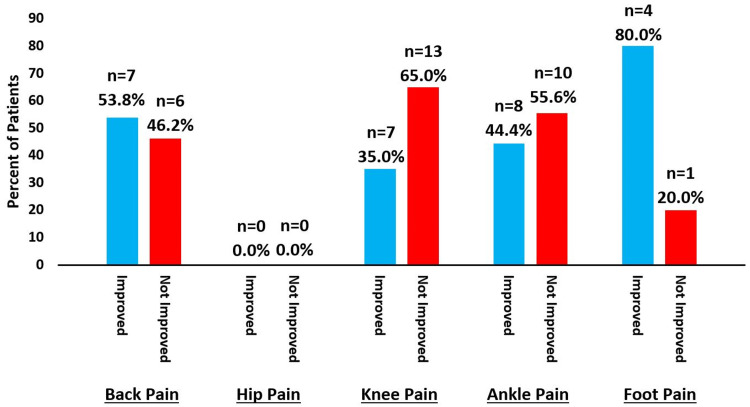
Initial locations of pain that improved over time. Baseline reported locations of pain compared to locations of pain at final follow-up.

Patients who initially reported zero locations of pain went on to report pain in an average of 2.4 locations at final follow-up. Those who reported one location at baseline reported three average locations at final follow-up, those who reported two locations at baseline reported 3.5 average locations at final follow-up, those who reported three locations at baseline reported two at final follow-up, and those who reported four locations at baseline reported on an average one location at final follow-up. The patients who reported five locations of pain did not have follow-up questions about locations of pain recorded. Of those with recorded follow-ups, 53.8% of patients with back pain at the initial visit, 35.0% of patients with knee pain, 44.4% of patients with ankle pain, and 80.0% of patients with foot pain improved at final follow-up, with patients who initially reported hip pain but did not have any recorded follow-up about locations of pain (Figure 2).

**Figure 3:**
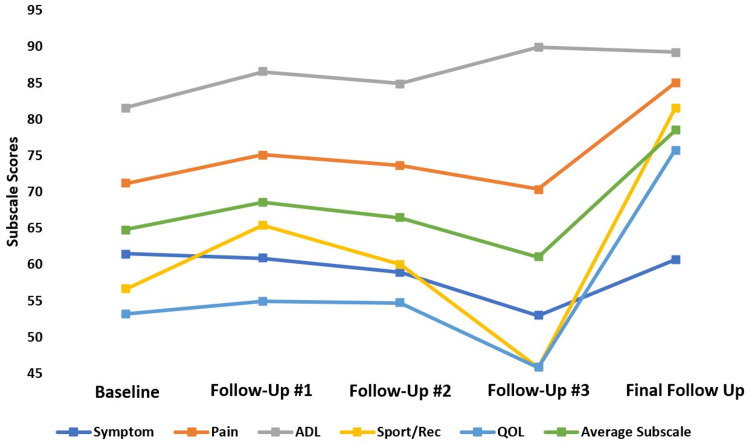
FAOS scores. Foot and Ankle Outcome Scores (FAOS) as reported by patients over time. Here 100 indicates no symptoms and zero indicates extreme symptoms. ADL: activities of daily living; Sport/Rec: sports and recreation; QOL: quality of life score

**Figure 4:**
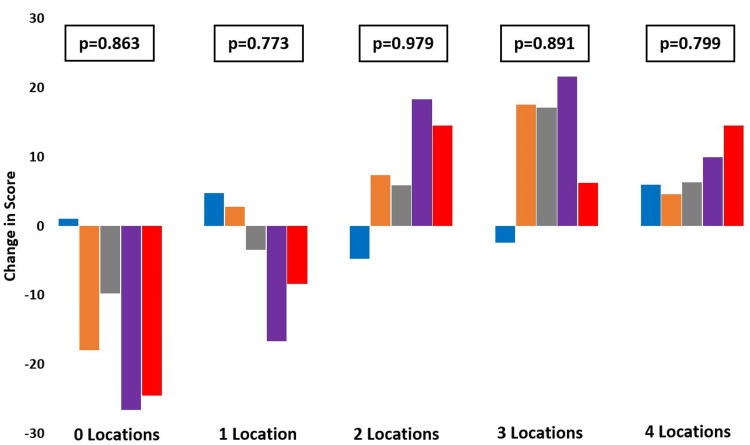
Changes in FAOS scores by initial presentation. Foot and Ankle Outcome Scores (FAOS) as reported by patients over time and their change from baseline to final recorded follow-up. Data are stratified by number of locations of pain reported at initial evaluation. Statistical analysis was performed using a one-way ANOVA test with p≤0.05 denoting significance (blue=symptoms score; orange=pain score; gray=ADL score; purple=Sports and Rec score; red=QOL score). ADL: activities of daily living; Sport and Rec: sports and recreation; QOL: quality of life

Overall, patients reported worsening symptom subscale scores before ending up with a score similar to their reported baseline. Pain subscale scores showed a decreasing amount of improvement compared to baseline scores over time; however, there was a large improvement in scores at the final follow-up (13.89 points). The activities of daily living (ADL) subscale score showed an overall improvement in scores over time, with a 7.64-point improvement at the final follow-up. The sports and recreation (Sport and Rec) and quality of life (QOL) subscale scores also showed a decreasing amount of improvement compared to baseline until there was a significant improvement in scores at the final follow-up (Sport and Rec=24.92 points and QOL=22.61 points) (Figure 3). While patient-reported sports and recreation subscale scores demonstrated a significant improvement at the final follow-up compared to baseline scores (p=0.02), all other scores did not significantly differ compared to baseline scores (Table 2). There was also no difference in symptom (p=0.863), pain (p=0.773), ADL (p=0.979), sports and recreation (p=0.891), or QOL (p=0.799) subscale scores from baseline to final follow-up between patients who reported differing numbers of locations of pain at initial evaluation (Figure 4).

**Table 2:** Changes in FAOS scores compared to baseline. Foot and Ankle Outcome Scores (FAOS) are reported by patients on a scale of zero to 100, where 100 indicating no symptoms and zero indicating extreme symptoms. Negative changes signify a decrease in scores (more extreme symptoms) while positive changes signify an increase in scores (less extreme symptoms). Wilcoxon signed-rank tests were used to compare each follow-up score with baseline score, with p≤0.05 denoting significance. ADL: activities of daily living; Sport/Rec: sports and recreation; QOL: quality of life

	Follow-Up 1		Follow-Up 2		Follow-Up 3		Final Follow-Up	
	Mean±SD (Range)	p-Value	Mean±SD (Range)	p-Value	Mean±SD (Range)	p-Value	Mean±SD (Range)	p-Value
Symptom	0.30±13.11 (-32.14 to 32.14)	1.00	0.89±19.21 (-17.86 to 46.43)	0.40	-4.17±5.12 (-10.71 to 1.79)	0.11	1.12±10.08 (-14.29 to 25.00)	0.90
Pain	7.75±19.71 (-33.33 to 58.33)	0.50	5.56±21.02 (-11.11 to 52.78)	0.78	11.11±6.00 (5.55 to 19.44)	0.18	17.19±29.32 (-36.11 to 77.78)	0.19
ADL	8.70±18.03 (-23.53 to 58.82)	0.63	4.60±10.72 (-14.71 to 26.47)	0.48	6.13±1.25 (4.41 to 7.35)	0.59	10.85±29.88 (-63.24 to 79.41)	0.50
Sports/Rec	13.54±22.20 (-15 to 85)	0.34	9.38±20.38 (-30 to 45)	0.73	-4.17±18.52 (-30 to 12.5)	0.29	27.5±29.26 (-35 to 70)	0.02
QOL	9.11±18.22 (-25 to 56.25)	0.45	17.19±25.34 (-18.75 to 50)	0.57	25±13.50 (6.25 to 37.5)	0.41	25.39±37.92 (-43.75 to 100)	0.08

Fifty-seven (91.9%) patients reported playing sports during their initial evaluation. In total, 37 (59.7%) patients participated in gym class, 11 (17.7%) in dance, and 26 (41.9%) in team sports. Twelve (19.4%) patients played soccer, eight (12.9%) played basketball, four (6.5%) played baseball, three (4.8%) ran (cross country and track), two (3.2%) played racket sports (tennis and squash), one (1.6%) played football, one (1.6%) wrestled, one (1.6%) played volleyball, one (1.6%) played hockey, and six (9.7%) played multiple sports. Additionally, six (9.7%) patients also reported participating in other activities (two in gymnastics, two in cheerleading, one in color guard, and one in ice skating). At their final follow-up, 12 of 26 (46.2%) patients reported being able to return to play in their desired sport, three (11.5%) patients reported not being able to return to play, and 11 (42.3%) declined to answer. Of the patients who were able to return to sports, nine initially presented with zero locations of pain, one with two locations, and two patients with three locations of pain.

## Discussion

While complaints of ankle pain, knee pain, and back pain are quite common in the pediatric population, these symptoms may be part of a predictable constellation of findings that include low back pain, flexible flat feet, Achilles contracture, and altered gait due to increased lateral subluxation of the patella and hip flexion. While selected reports do exist about a correlation between pes planus, knee pain, and back pain, there is still no data published on the entire above constellation of symptoms [1-3,10]. This report details our cohort of patients who presented with back, hip, knee, ankle, or foot pain and their clinical course.

Most of our patients complained of lower back pain and medial patellar knee pain at initial evaluation. However, when examined thoroughly, they were found to have the entire described constellation of findings. While not described in the pediatric literature, there is published literature about a correlation between back pain, knee pain, and flatfeet [11,12]. Heightened awareness of the change in kinematics caused by flexible pes planus is needed, as this can cause a constellation of lower extremity symptoms in multiple anatomic locations [13,14]. This change in lower extremity kinematics is probably due to the well-documented change in gait mechanics in patients with pes planus [15,16]. Even if the other findings did not cause pain or symptoms initially, they were discovered before they became troublesome, highlighting the importance of a thorough physical examination. Given the proper treatment (conservative or surgical management based on clinical judgment in the face of severity of physical examination findings), slightly more than half of back complaints and less than half of knee complaints improved at final follow-up. Less than half of ankle pain also improved at the final follow-up. Statistical analysis of patients who underwent conservative versus surgical management was not undertaken due to low cohort numbers.

Previous studies have also detailed the lower quality of life outcome scores associated with more severe pediatric flatfoot [17]. While all patients in our study on average had a higher FAOS score signifying improving symptoms at the final follow-up (most notably in Sport and Rec and QOL scores), this improvement was not statistically significant. The decision to record FAOS scores for each patient, instead of other scores, was secondary to the fact that each patients’ treatment centered around the conservative or surgical intervention of Achilles contracture and pes planus as necessary. The authors believe that the lack of a statistically significant improvement is secondary to low patient numbers in our cohort, and if diagnosed early and treated properly, these patients do improve over time. This improvement is further emphasized by the fact that 12 out of 15 patients who responded noted that they were able to return to play their desired sports.

The lack of difference in improvement based on the initial presentation is an important finding that must be highlighted. No matter how many locations of pain a patient presented with, they had similar improvement in FAOS scores over time. In fact, patients on average reported more locations of pain at follow-up visits compared to their initial examination, indicating that they did develop complaints in a predictable pattern after their diagnosis was made. This seems to indicate that while early diagnosis is important, proper treatment centered around braces, orthotics, stretching, and strengthening can improve symptoms in these patients no matter when and how they present. The increased number of locations of pain did not correlate to the improvement seen in FAOS scores. There was no qualification of severity of pain or other outcome scores that integrate different anatomic locations of symptoms used in this cohort.

This retrospective study documents a series of patients who present with a previously undescribed triad of symptoms as well as their clinical progression over time. However, this study does have its weaknesses. While this series only includes 64 patients, it does provide a solid foundation for the further exploration of this triad. Not every patient followed up for the entire two-year period, leaving an unequal number of scores at each time point and possibly skewing the data. Not every patient followed up at uniform times. The decreasing number of patients presenting for follow-up also curtailed the ability to run further stratified analysis on patient groups. Further exploration of this triad should build off this report, using subjective and objective outcome measures that encompass all anatomic locations of symptoms to document clinical progression and treatment options. Evaluation of additional confounding anatomical anomalies and variations may include femoral and tibial torsion, ligament laxity, genu recurvatum, valgus and varus may be of importance in future studies.

## Conclusions

Complaints of back, hip, knee, ankle, or foot pain in the pediatric population can be early markers for a constellation of conditions that include low back pain, flexible flat feet, Achilles contracture, and altered gait due to increased lateral subluxation of the patella and hip flexion. Early diagnosis of the entire constellation of symptoms can be made using a thorough history and physical exam coupled with an awareness of this constellation. When diagnosed and treated appropriately, these patients do clinically improve over time with return to sport possible.
